# Diagnostic evaluation and medication usage in a cohort of subjects with juvenile dermatomyositis from the CARRAnet registry

**DOI:** 10.1186/1546-0096-10-S1-A64

**Published:** 2012-07-13

**Authors:** Angela B Robinson, Mark F Hoeltzel, Ann M Reed, Adam Huber, Brian M Feldman

**Affiliations:** 1 CARRAnet Investigators; 2 Children's Mercy Hospital, Kansas City, MO, USA; 3 IWK Health Centre, Halifax, NS, Canada; 4 Mayo Clinic, Rochester, MN, USA; 5 Rainbow Babies and Childrens Hospital, Cleveland, OH, USA; 6 The Hospital for Sick Children, Toronto, ON, Canada

## Purpose

Juvenile dermatomyositis (JDM) is a rare disease which has been difficult to evaluate objectively due to the low incidence of disease. The Childhood Arthritis and Rheumatology Research Alliance (CARRA) initiated a multi-center observational registry to create a clinical database for the major rheumatic diseases of childhood, including JDM. Initial data from the JDM cohort (prevalent and incident cases) enrolled in the first 7 months of this ongoing study are evaluated here.

## Methods

Children under 21 yrs with onset of JDM prior to 16 yrs were included, and subjects or their guardians were consented for the study. IRB approval was obtained at each enrolling site. JDM was diagnosed by modified Bohan and Peter criteria. Clinical data were collected from the subjects, guardians, and providers using both general and JDM-specific case report forms at the time of enrollment. Data regarding demographics, diagnostic assessment, and medication exposure were collected. Data were pooled and stored in a secure centralized database and de-identified prior to analysis.

## Results

Between May 28, 2010 and December 28, 2010, 102 subjects meeting modified criteria for JDM were enrolled from 23 sites in the U.S. Diagnostic studies commonly used include electromyography (EMG), muscle biopsy, and magnetic resonance imaging (MRI). Overall, MRI was more likely than EMG or muscle biopsy to show abnormalities. (Table 1) 48 of subjects had 2 or more studies performed and 54.2% of these subjects reported at least 1 negative study. In terms of medications, 100% of subjects have been exposed to corticosteroids during their course of treatment, and 97% of subjects have been exposed to methotrexate, suggesting that these medications are almost universally prescribed for JDM. Medication history in order of frequency of usage is shown in Table 2.

**Table 1:** Evaluation of diagnostic modalities in JDM

	N (%)
Muscle biopsy (n=89)	
Performed	44 (49.4)
Consistent with JDM	29 (65.9)
EMG (n=86)	
Performed	36 (41.9)
Consistent with JDM	25 (69.4)
MRI (n=87)	
Performed	74 (85.1)
Consistent with JDM	61 (82.4)
Combination of ≥2 studies (n=91)	
Performed	48 (52.7)
2 studies consistent with JDM	26 (54.2)
A study consistent with JDM	22 (45.8)

**Table 2:** Medication usage with the CARRAnet JDM cohort (N=102)

	Current use	Previous use	Never used	Missing
All corticosteroids	51 (52.6%)	46 (47.4%)	0 (0.0%)	5
Daily corticosteroids	50 (51.5%)	46 (47.4%)	1 (1.1%)	5
Methotrexate	65 (68.4%)	27 (28.4%)	3 (3.2%)	7
Pulse corticosteroids	4 (4.2%)	48 (50.5%)	43 (45.3%)	7
Hydroxychlorquine	39 (41.1%)	17 (17.9%)	39 (41.1%)	7
Intravenous gammaglobulin	24 (24.5%)	24 (24.5%)	50 (51.0%)	4
Mycophenolate mofetil	13 (13.7%)	5 (5.3%)	77 (81.1%)	7
Cyclosporine A	4 (4.2%)	5 (5.3%)	86 (90.5%)	7
Rituximab	1 (1.0%)	7 (7.1%)	90 (91.8%)	4
Cyclophosphamide	0 (0.0%)	2 (2.1%)	93 (97.9%)	7

## Conclusion

MRI was the most common diagnostic modality used and was the most likely to show abnormalities consistent with JDM. The false negative rates for MRI, EMG, and muscle biopsy alone were higher than expected if ascertainment is correct. Corticosteroids and methotrexate appear to be standard first line medications used by US pediatric rheumatologists for JDM. Pulse corticosteroids, intravenous gammaglobulin, and hydroxychloroquine have been used by about half of subjects and further investigation as to which subgroups receive these medications is warranted.

## Disclosure

Angela B. Robinson: None; Mark F. Hoeltzel: None; Ann M. Reed: None; Adam Huber: None; Brian M. Feldman: Baxter, 2, Bayer, 2, Novartis Pharmaceuticals Corporation, 6; Juvenile Myositis CARRA Subgroup: None.

